# Impact of applying different levels of threshold-based artifact correction on the processing of heart rate variability data in individuals with temporomandibular disorder

**DOI:** 10.1038/s41598-024-76287-z

**Published:** 2024-10-19

**Authors:** Rodrigo Costa Cutrim, Aldair Darlan Santos-de-Araújo, Cassius Iury Anselmo-e-Silva, Edna Cristina Pinheiro Ferreira, Tatyana Santana de Azevedo Silva, Almir Vieira Dibai-Filho, Daniela Bassi-Dibai

**Affiliations:** 1 Postgraduate Program in Dentistry, Ceuma University, São Luís, MA Brazil; 2 Department of Physical Therapy, Federal University of São Carlos, São Carlos, SP Brazil; 3 Postgraduate Program in Management of Health Programs and Services, Ceuma University, São Luís, MA Brazil; 4 Postgraduate Program in Adult Health, Federal University of Maranhão, São Luís, MA Brazil; 5 Department of Physical Therapy, Ceuma University, São Luís, MA Brazil; 6 Postgraduate Program in Programs Management and Health Services, Ceuma University, Rua Josué Montello, 1, Jardim Renascença, São Luís, 65075-120 MA Brazil

**Keywords:** Temporomandibular disorder, Autonomic nervous system, Heart rate variability, Computational biology and bioinformatics, Data acquisition, Data processing

## Abstract

Although heart rate variability (HRV) is a valid method to evaluate the behavior of the autonomic nervous system in individuals with temporomandibular disorder (TMD), the measurement can easily be biased by factors involving the analysis methodology, such as the removal of artifacts. Therefore, the objective of this investigation is to evaluate the impact of using different levels of threshold-based artifact correction to process HRV data in individuals with TMD. This cross-sectional observational study. Adults aged 18 to 55 years old with a diagnosis of myogenic TMD, score ≥ 50 on the Fonseca Anamnestic Index (FAI) and pain ≥ 3 on the Numerical Pain Scale (NPS) participated. The HRV was registered in the supine position (short-term) using a Polar S810i. Kubios software was used for HRV analysis using all filters. One-way ANOVA with Tukey-Kramer post-hoc was used to test the differences in HRV using the different Kubios Software artifact correction filters. The effect size was calculated based on the Cohen d. The very strong filter was statistically different (*p* < 0.05) compared to the no filter in all overview and time domain variables. In the frequency domain, the variables VLF, LF, HF and Total Power showed statistical differences (*p* < 0.05) when using the very strong filter. The same occurred with the variables SD1, SD2 and DFA α2 of the non-linear analysis (*p* < 0.05). The most restrictive filter of the Kubios software (very strong) significantly impacts the quantification of HRV parameters in individuals with TMD.

## Introduction

The proof of the association between autonomic nervous system (ANS) and TMD has heightened the curiosity of clinicians and scientists to explore the impact of TMD on different outcomes involving autonomic balance, in addition to understanding the physiological mechanisms that justify such an association^1–3^. At first, it is important to highlight the causal relationship between the appearance of TMD and ANS dysfunction and vice versa^4^. Physiologically, the main mechanism that can minimally justify this interaction is associated with the chronic and persistent pain faced by this population, which ends up triggering high levels of stress and anxiety, among other factors that compromise the integrity of the ANS^4^. An important aspect that deserves to be highlighted is the central sensitization present in this population manifested in the form of hypersensitivity to pain due to functional and structural changes in the somatosensory cortex and its association with disorders involving the ANS^5,6^.

When it comes to the integrity of the ANS, some widely known methods are used to investigate the balance between the sympathetic and parasympathetic nervous systems in TMD^1,2,5,7^. Among them, HRV has endorsed numerous scientific evidence due, mainly, to its non-invasive characteristic for acquiring information on cardiovascular health, more precisely on the autonomic axis that involves the brain and heart^8^. Although HRV is a valid method capable of reflecting and mathematically representing the nature of the ANS, the measurement can easily be biased by several factors involving individual (age and gender), physiological (stress and breathing), environmental (temperature and external noise), lifestyle (diet and cigarette use), psychological (anxiety and depression) and technological (accuracy of devices and analysis methodologies)^9^.

Despite this last factor, different methodologies for analysis, identification and removal of artifacts and selection of RR intervals, that is, the stretch of best stationarity of the HRV signal, have been reported in the literature^10^. The heterogeneity of these aspects, combined with the fact that HRV is an evaluator-dependent outcome, can generate conflicting and dubious results, since uncontrolled biases negatively influence the interpretation of the outcome from the beginning of the collection^9^. One of the most used software for HRV analysis has some pre-processing tools that automatically detect noise and offers a package of corrective filters capable of removing artifacts identified throughout the HRV signal, which basically uses interpolations to replacement of compromised RR intervals^11^. Despite facilitating the removal of artifacts, the choice of filter is based on subjective decisions and the impact of using these filters was previously explored in some populations whose results were shown to be influenced by the choice between them^12,13^.

The biggest concern regarding the use of these filters is linked to the fact that they clinically influence the results, generate erroneous interpretations and decision-making^12,13^, especially when we consider the increasing rise in the use of this outcome in the assessment of ANS in individuals with TMD^1,14,15^. Given this context, the objective of this investigation is to evaluate the impact of using different levels of threshold-based artifact correction to process HRV data in individuals with TMD. Our hypothesis is that more restrictive filters (strong and very strong) have a significant impact on the quantification of HRV variables, generating important clinical implications.

## Methodology

### Study design and ethical aspects

This is an observational cross-sectional study. The research was carried out at the Universidade CEUMA and the project was previously approved by the institution’s Research Ethics Committee (protocol number 5.674.373). The study followed the recommendations of the Declaration of Helsinki. Recruit your volunteers through social media and word-of-mouth. They underwent evaluation with the dentist.

Previously, all volunteers with the potential to be included in the study were informed about the research objectives and the methods to be used. Once the volunteer agreed to participate in the investigation, the free and signed consent form was collected.

### Participants

Adults aged 18 to 55 with a confirmed diagnosis of myogenic TMD, indicated by a score of 50 or more on the Fonseca Anamnestic Index (FAI)^16^ and a pain level of 3 or above on the Numerical Pain Scale (NPS)^17^, were included. Individuals were excluded if they had clinical diagnoses of rheumatic, cardiovascular, metabolic, or respiratory conditions; wore full or partial dental prostheses; had systemic neuromuscular disorders; had a history of facial and/or temporomandibular joint trauma; experienced joint dislocation; had cancer; suffered from active inflammatory or infectious diseases; were diagnosed with fibromyalgia; were smokers; or had any other condition that would prevent them from undergoing the proposed evaluations.

### Numerical Pain Scale (NPS)

The NPS^17^ is a simple and easy-to-use measure consisting of a sequence of numbers from 0 to 10. In this scale, the value 0 indicates “no pain” and the value 10 represents “the worst pain imaginable.” Thus, the volunteers rated their pain based on these parameters. Pain intensity was assessed with the individual at rest.

### Fonseca Anamnestic Index (FAI)

The FAI was used to assess the presence and severity of TMD symptoms. This is a simple and easy-to-understand instrument, allowing patients to fill it out themselves, if necessary, without compromising the quality of the assessment. However, in this investigation, the FAI was carried out through an interview by a single previously trained examiner. Participants answered a total of 10 questions, with three response options: “yes” (score: 10), “no” (score: 0) and “sometimes” (score: 5). The final score of the instrument is determined by the sum of the scores of all items, allowing the following classifications: absence of signs and symptoms of TMD (0–15 points), mild TMD (20–45 points), moderate TMD (50–65 points) and severe TMD (70–100 points)^18–22^.

### Central Sensitization Inventory (CSI)

The CSI which has been validated for the Brazilian population^23^, was utilized to evaluate central sensitization symptoms in participants with chronic pain. A single examiner conducted the questionnaire through an interview. It consisted of 25 questions about symptoms or health situations in the participant’s daily life, with five possible responses for each question: “never” (score: 0), “rarely” (score: 1), “sometimes” (score: 2), “often” (score: 3), and “always” (score: 4). The total score ranges from 0 to 100, with a previously established cutoff value of 30 points or more to address the possible presence of central sensitization^24^.

### R-R intervals recordings – heart rate variability

Standardized procedures for collecting biological signals for subsequent analysis of heart rate variability have been previously published^1^.

### Data processing

We used the Kubios HRV standard analysis software (MATLAB, version 3.5, Kuopio, Finland) to process the HRV data^11^. To this end, the stretch with the greatest stationarity lasting 5 min, that is, short-term analysis, was selected for analysis. The choice of this section followed some previously established criteria: (1) absence of significant R–R interval outliers (i.e., R–R intervals markedly higher or lower than the overall R–R signal, as determined through visual inspection by the researcher); (2) equidistance of R–R intervals; and (3) Gaussian distribution of R–R intervals and heart rate distribution graphs^12^.

All corrective filters from the Kubios software were used to evaluate the results^11^. All corrective filters available in the Kubios software were used to evaluate the results. Basically, the correction algorithm makes an RR interval value comparison with a local average interval. The local mean is obtained by filtering the median of the RR interval time series, and therefore, single outliers in the RR interval time series do not affect the local mean. If an RR interval differed from the local mean by more than a specified threshold value, the interval was identified as an artifact and marked for correction. The threshold value can be selected between: (1) None (no correction is performed); (2) Very low: 0.45 s (threshold in seconds); (3) Low: 0.35 s; (4) Medium: 0.25 s; (5) Strong: 0.15 s; and (6) Very strong: 0.05 s. To give an example, the “average” correction level will identify all RR intervals greater or less than 0.25 s compared to the local average. The correction is made by replacing the identified artifacts with interpolated values ​​using a cubic spline interpolation.

HRV was analyzed using overview parameters, linear statistical measures (time and frequency domain) and through non-linear statistical measures. In the overview, the following variables were used: parasympathetic nervous system (PNS) index, sympathetic nervous system (SNS) index and stress index. In the time domain, the following variables were analyzed: the average interval between R-waves (Mean RR) in milliseconds (ms); ms: milliseconds; standard deviation of all N-N normal intervals (SDNN) in ms; the average of heart rate (Mean HR) in beats per minute (bpm); square root of the mean squared differences of successive RR (RMSSD) in ms; number of interval differences of successive NN intervals greater than 50 ms divided by the total number of NN intervals (pNN50) in percentage (%); integral of the density of the RR interval histogram divided by its height (RR Tri); baseline width of the RR interval histogram (TINN) in ms. For frequency domain analysis, the following variables were evaluated: very-low-frequency band (0.0033–0.04 Hz) in milliseconds squared (ms^2^); LF: low frequency band (0.04–0.15) in ms^2^ and n.u.; HF: high frequency band (0.15–0.40) in ms^2^ and n.u.; total power in ms^2^ and LF/HF ratio^8,25^.

For non-linear analysis, the following variables were considered: standard deviation perpendicular to the line of identity (SD1) in ms; standard deviation along the line of identity (SD2) in ms; ratio of SD2-to-SD1 (SD2/SD1); approximate entropy (ApEn); sample entropy (SampEn); detrended fluctuation analysis which describes short-term fluctuations (DFA α1); detrended fluctuation analysis, which describes long-term fluctuations (DFA α2)^8,25^.

### Statistical analysis

Data are presented as mean and standard deviation or absolute values and percentages of occurrence when appropriate. The Shapiro-Wilk test was used to verify the normality of the data. When the data met parametric assumptions, One-way ANOVA with Tukey-Kramer post-hoc was used to test the differences in HRV using the different Kubios Software artifact correction filters (none, very low, low, medium, strong and very strong).

For data with non-parametric distribution, the Friedman test with Dunn’s post hoc was used. χ^2^ test was used to compare categorical variables. The effect size was calculated based on the Cohen d, according to the website: <https://www.psychometrica.de/effect_size.html>. It was considered the following interpretation of the d value: 0.2 (small), 0.5 (moderate) and > 0.8 (large effect size)^26^.

All analyzes were performed using GraphPad Prism software (version 8.0.1 for Windows, GraphPad Software, San Diego, California USA). The probability of type 1 error occurrence was established at 5% for all tests (*p* < 0.05).

## Results

Thirty-six volunteers were included following the research inclusion criteria. Data with sample characterization can be viewed in (Table 1). Briefly, the mean age was 34.25 ± 8.64. The sample was mainly composed of female individuals (73%). The participants’ mean pain intensity according to the ANS was 6 ± 1. Half of the sample (50%) had moderate TMD according to the FAI. Finally, the CSI presented a mean value that indicates the presence of central sensitization when considering the previously described cutoff point > 30 points.

**Table 1:** Personal and clinical characteristics of the study sample (*n* = 36).

Variables	Mean (SD) ou absolute value (%)
Age (years)	34.25 ± 8.64
Gender	
Male n (%)	10 (27)
Female n (%)	26 (73)
Weight (kg)	70.50 ± 13.85
Height (m)	1.63 ± 0.08
BMI (kg/m²)	25.98 ± 3.60
Pain - AVS	6 ± 1
Fonseca Anamnestic Index	55.00 ± 9.28
Mild TMD	9 (25)
Moderate TMD	18 (50)
Severe TMD	9 (25)
CSI	37.72 ± 11.49
Respiratory rate at rest (minute)	16 ± 3

SD: standard deviation; BMI: body mass index; kg: kilos; m: meter; AVS: analogic visual scale. TDM: temporomandibular disorder.

**Fig. 1:**
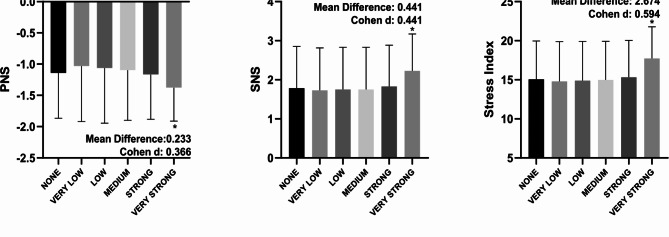
Illustration of heart rate variability indices in the overview parameters using different filters of Kubios software. PNS: parasympathetic nervous system; sympathetic nervous system. *significant difference in relation to none; Statistical significance (*p* < 0.05) for One-way ANOVA (Tukey-Kramer post-hoc) or Friedman test (Dunn’s post-hoc).

**Fig. 2:**
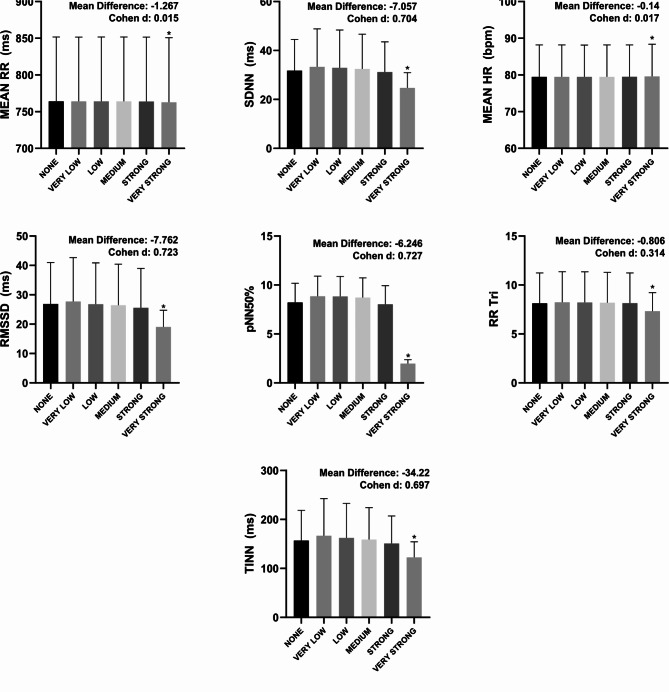
Illustration of heart rate variability indices in the time domain using different filters of Kubios software. Mean RR: the average interval between R-waves; ms: milliseconds; SDNN: standard deviation of all N-N normal intervals; Mean HR: the average of heart rate; bpm: beats per minute; RMSSD: square root of the mean squared differences of successive RR; pNN50: number of interval differences of successive NN intervals greater than 50 ms divided by the total number of NN intervals; %: percentage; RR Tri: Integral of the density of the RR interval histogram divided by its height; TINN: baseline width of the RR interval histogram. ^*^significant difference in relation to none; Statistical significance (*p* < 0.05) for One-way ANOVA (Tukey-Kramer post-hoc) or Friedman test (Dunn’s post-hoc).

**Fig. 3:**
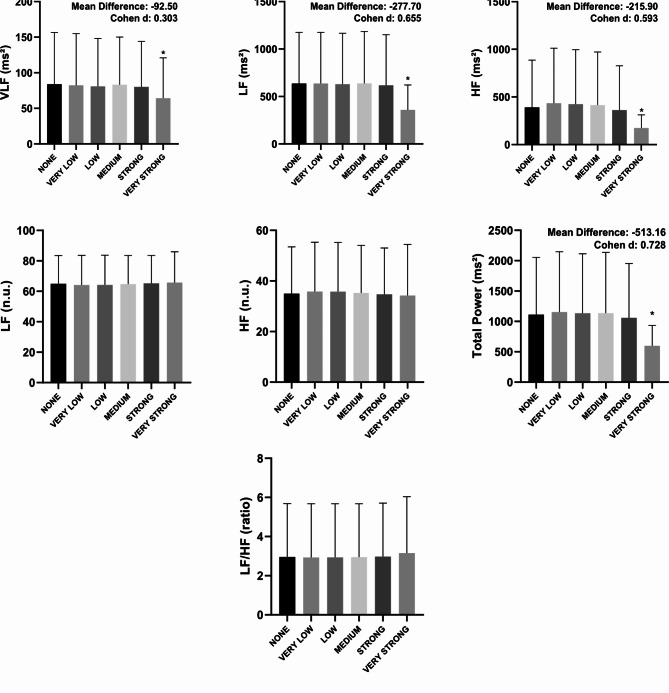
Illustration of heart rate variability indices in the frequency domain using different filters of Kubios software. Values are mean ± SD. VLF: very-low-frequency band (0.0033–0.04 Hz); HF: high frequency band (0.15–0.40); LF: low frequency band (0.04–0.15); ms²: milliseconds square; n.u.: normalized units. ^*^significant difference in relation to none. Statistical significance (*p* < 0.05) for One-way ANOVA (Tukey-Kramer post-hoc) or Friedman test (Dunn’s post-hoc).

**Fig. 4:**
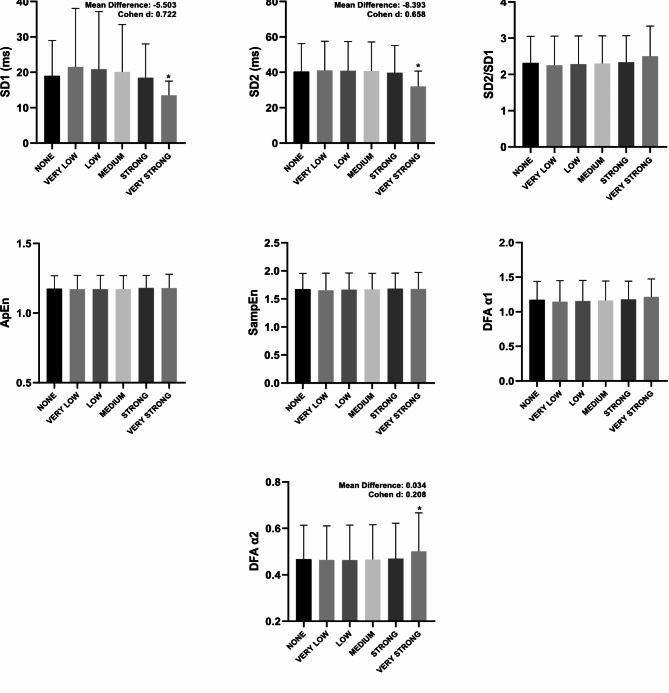
Illustration of heart rate variability indices in the non-linear analysis using different filters of Kubios software. Values are mean ± SD. SD1: standard deviation perpendicular the line of identity; SD2: standard deviation along the line of identity; SD2/SD1: ratio of SD2-to-SD1; ApEn: approximate entropy; SampEn: sample entropy; DFA α1: detrended fluctuation analysis which describes short-term fluctuations; DFA α2: detrended fluctuation analysis, which describes long-term fluctuations.^*^significant difference in relation to none. Statistical significance (*p* < 0.05) for One-way ANOVA (Tukey-Kramer post-hoc) or Friedman test (Dunn’s post-hoc).

In Fig. 1, the very strong filter showed a statistical difference (*p* < 0.05) when compared to the none filter in all overview variables (PNS, SNS and Stress Index) with Cohen d varying between small to moderate. According to Fig. 2, in all time domain variables, the very strong filter also showed a statistical difference (*p* < 0.05) when compared to the none filter. The effect size behavior according to Cohen d was similar to the overview variables: they ranged from small to moderate effect size; with the exception of the Mean RR variable, which presented an effect size < 0.2. The Fig. 3 illustrates the comparisons between the different filters in the frequency domain variables. Only in the variables VLF (ms²), LF (ms²), HF (ms²) and Total Power (ms²) did the very strong filter prove to be different when compared to none with Cohen d varying between small to moderate. As for the non-linear analysis in Fig. 4, only in the variables SD1 (ms), SD2 (ms) and DFA α2 did the very strong filter show a statistical difference (*p* < 0.05) with an effect size ranging from small to moderate. In the figures previously mentioned, the respective values ​​of the average differences calculated between the very strong and none filters can be seen.

## Discussion

Our main finding is: (1) the most restrictive filter, that is, very strong, significantly impacts the HRV variables in the overview, linear analysis (time domain and frequency domain) and non-linear analysis, in individuals with TMD, confirming our previously outlined hypothesis; (2) when considering Cohen’s d values, such differences can generate significant clinical impacts due to the nature of the differences observed between the comparisons.

HRV as an outcome for assessing ANS in this population was recently introduced^15^. In fact, the scientific field that addresses aspects involving TMD and HRV is scarce and lacks evidence to attest to its discriminatory capacity in different scenarios involving the pathology. Despite this, considering the current evidence involving the relationship between the ANS and TMD, we need to highlight, initially, the autonomic imbalance reflected in increased expression of sympathetic modulation^1,4,14,15^. Among the complexity of the nature of TMD, one of the aspects that is directly related to this imbalance is the clinically characteristic pain of the pathology^4^. The influence of chronic pain in musculoskeletal dysfunctions on autonomic control has been previously scientifically proven and the main mechanism behind it is explained in light of the impact of painful nociception faced by individuals and activation of the sympathetic nervous system^27^.

Recently, we observed that individuals with TMD, when compared to a control group without TMD, present a decreased short-term HRV (5 min) in rest reflected in greater sympathetic activation and less parasympathetic modulation^1^. Additionally, Eze-Nliam et al.^15^, when observing whether patients with painful myofascial TMD had decreased nocturnal HRV when compared to a healthy, pain-free control group, came to the conclusion that the group affected by TMD had losses in nocturnal HRV, with greater expression of sympathetic nervous system. Despite the limitation of the methodological design of both studies, the literature had previously confirmed the dysautonomia observed in this population and some hypotheses were raised and defended^4^.

Parallel to our findings, we can highlight some evidence that has attested to the impact of HRV RR interval editing and selection techniques, including the use of filters available in the Kubios software, in the clinical and scientific context, especially in aspects involving prognoses and decision-making due to the cut-off points established for different outcomes^12,13,28^. Previously, Alcantara et al.^12^, while investigating the impact of different Kubios filters on quantifying HRV-derived parameters from short-term recordings in three independent human cohorts (overweight/obese children, young adults, and middle-aged adults), observed that the use of certain Kubios filters has an important impact on the quantification of parameters derived from HRV, especially in children and young adults who, due to the fact that they have been more affected by less restrictive filters, that is, of lower intensity (very low, low and medium).

Our results are similar to the study by Aranda et al.^13^. The authors observed that the use of the very strong filter showed significant differences in short-term HRV (10 min) in the time domain and frequency domain when compared to other filters in a group of athletes. In patients with exacerbated and non-exacerbated COPD, when strong and, especially, very strong filters are used in quantifying short-term HRV at rest, the R-R intervals of the section selected for analysis may suffer severe interpolations lead to implications for the interpretation of results^29^.

## Clinical relevance

Clinically, the results found here suggest that the use of artifact correction filters in the population studied here should be used with caution, especially or more restrictively, since their use may generate hasty conclusions. Regardless of the methodological nature of future studies involving HRV in individuals with TMD, or their use, whether for scientific or clinical purposes, the researchers and clinicians involved need to describe whether or not they use interpolation methods to correct artifacts so that scientific reproducibility is guaranteed and the interpretation of the results does not become misleading.

## Limitations

First, the sample was composed mostly of women, which may limit the generalization of the findings to men or other clinical populations. In addition, the sample is relatively small. Future studies may be needed to confirm the nature of the results found here in a significantly larger sample and stratified by sex. The evaluation of cardiac autonomic modulation by HRV in individuals with TMD is still very elementary and the literature is scarce and limited. This limited us to project the results using literature that addressed HRV in individuals with TMD. However, this study, in addition to encouraging the scientific community and researchers involved with this population to use HRV as an outcome and clarify the relationship between cardiac autonomic dysfunction and TMD, opens the possibility of further exploration in this field of investigation.

## Conclusion

The very strong filter, when used, interpolates the values ​​of the HRV parameters and underestimates their estimates, which can generate biased results that do not reflect the reality of cardiac autonomic modulation in individuals with TMD and impact their clinical interpretation.

## Data Availability

The set of data generated and/or analyzed during the present study are available through the corresponding author upon reasonable request.
